# Floral micromorphology of the Australian carnivorous bladderwort *Utricularia dunlopii*, a putative pseudocopulatory species

**DOI:** 10.1007/s00709-015-0900-8

**Published:** 2015-10-26

**Authors:** Bartosz J. Płachno, Małgorzata Stpiczyńska, Piotr Świątek, Kevin L. Davies

**Affiliations:** 1 Department of Plant Cytology and Embryology, Jagiellonian University in Kraków, 9 Gronostajowa St., 30-387 Kraków, Poland; 2 University of Warsaw, Faculty of Biology, Botanic Garden Al. Ujazdowskie 4, 00-478 Warsaw, Poland; 3 Department of Animal Histology and Embryology, University of Silesia, 9 Bankowa St., 40-007 Katowice, Poland; 4 School of Earth and Ocean Sciences, Cardiff University, Main Building, Park Place, Cardiff, CF10 3AT UK

**Keywords:** Bladderwort, Carnivorous plant, Lentibulariaceae, Micromorphology, Osmophore, Pollination, Sect. *Pleiochasia*, Sexual deceit, Ultrastructure

## Abstract

Flowers of sexually deceptive taxa generally possess a set of morphological and physiological characters that mimic their insect pollinators. These characters often include a specific insect-like floral configuration, together with scent glands (osmophores) that produce fragrances which chemically resemble insect sex pheromones. Furthermore, these flowers tend not to produce pollinator food rewards. According to some authors, flowers of the Australian bladderwort *Utricularia dunlopii* (and species of the *Utricularia capilliflora* complex) resemble insects, and pollination perhaps occurs by pseudocopulation. The aims of this paper are to compare the structure and distribution of floral glandular trichomes in the Australian carnivorous plant *U. dunlopii* with those of closely related species assigned to the same section and to discuss their putative function. Floral tissues of *U. dunlopii* P. Taylor, *Utricularia paulinae* Lowrie, *Utricularia dichotoma* Labill. and *Utricularia uniflora* R.Br. (section *Pleiochasia*) were investigated using light microscopy, scanning electron microscopy, transmission electron microscopy and histochemistry. In *U. dunlopii*, two long, erect, filiform appendages arising from the upper lip of the corolla, together with three arising from the lower lip, bear numerous glandular trichomes that may function as osmophores. In other species, such as *U. uniflora* and *U. paulinae*, glandular papillae on the corolla palate may also function as osmophores. The floral anatomical and morphological organisation of *U. dunlopii* differs from that of the other investigated species, indicating that its insect pollinators are also likely to differ. Morphological and ultrastructural observations, while generally contributing to our understanding of the flower of *U. dunlopii*, do not refute the possibility that pollination here may occur by pseudocopulation. Further field-based investigations, however, are now necessary to test this hypothesis.

## Introduction

The carnivorous genus *Utricularia* L. (bladderworts, family Lentibulariaceae) contains about 230 species (Taylor 1989; Fleischmann 2012; Jobson 2013) and can be divided into three monophyletic subgenera, namely, *Polypompholyx*, *Bivalvaria* and *Utricularia sensu* Müller and Borsch (2005). *Utricularia* is cosmopolitan and is represented by free-aquatic, affixed-aquatic, rheophytic, lithophytic, terrestrial, epiphytic and even aquatic-epiphytic species (Taylor 1989; Guisande et al. 2007).

The basic corolla structure of most species of *Utricularia* is relatively uniform and bilabiate, with the lower lip consisting of three fused petals expanded to form a corolla palate located at the entrance to the spur, and the upper lip formed of two fused petals. However, their flowers are morphologically very diverse (Taylor 1989), with the size of the corolla ranging from about 1 to 100 mm. The smallest flowers occur in *Utricularia simmonsii* Lowrie, Cowie & Conran (Lowrie et al. 2008; Lowrie 2013), and the largest in epiphytic and aquatic–epiphytic species which grow in the central ‘urns’ or water-filled receptacles formed by the bases of the leaves of bromeliads (Taylor 1989; Guisande et al. 2007). A floral spur is present in most *Utricularia* spp. (Taylor 1989) but is absent in others, e.g. *U. simmonsii* (Lowrie et al. 2008; Lowrie 2013). Only a few species have been examined for nectar production. Spur nectar occurs in extremely small volumes but has high sugar concentrations (Hobbhahn et al. 2006) or may be entirely lacking, as was reported for *Utricularia alpina* Jacq. (Jérémie 1989). Hobbhahn et al. (2006) showed that more than 50 species of bees, butterflies, moths, hawk-moths and dipterans visited the flowers of three terrestrial species from the Indian Western Ghats, namely, *Utricularia albocaerulea* Dalzell, *Utricularia purpurascens* J. Graham and *Utricularia reticulata* Sm. Ornithophily may occur in some species of *Utricularia* assigned to section *Orchidioides* A.DC., the pollinators here being hummingbirds (Taylor 1989). Recently, it was shown that the relatively large flowers of the Brazilian species *Utricularia reniformis* A.St.-Hil. are pollinated by species of the bee genera *Xylocopa* and *Bombus* (Clivati et al. 2014).

Five species of the subgenus *Polypompholyx sensu* Müller and Borsch (2005) sect. *Pleiochasia* Kamieński, namely, *Utricularia dunlopii* P. Taylor, *Utricularia capilliflora* F. Muell, *Utricularia dunstaniae* F. E. Lloyd, *Utricularia antennifera* P. Taylor and *Utricularia lowriei* R.W. Jobson, each with flesh-coloured flowers, have erect, filiform appendages to the petals. These characters are quite unusual for the genus as a whole. In *U. lowriei*, *U. antennifera* and *U. dunstaniae*, these antenna-like appendages arise from the lower lip of the corolla, whereas in *U. capilliflora* and *U. dunlopii*, they arise from the upper lip of the corolla (Taylor 1989; Jobson 2013; Lowrie 2013). However, it would appear that those species having upper corolla appendages (assigned to the *U. capilliflora* complex) have arisen twice independently (Reut and Jobson 2010) and, according to Lowrie (1995), flowers of these species much resemble insects (sexual floral mimicry), suggesting that pollination may occur by pseudocopulation.

Pollination mechanisms involving sexual mimicry are known to occur in many different genera of Orchidaceae (e.g. van der Pijl and Dodson 1966; Dafni and Bernhardt 1989; Servettaz et al. 1994; Peakall and Beattie 1996; Kores et al. 2001; Singer 2002; Singer et al. 2004; Ascensao et al. 2005; Blanco and Barboza 2005; Ciotek et al. 2006; Flach et al. 2006; Phillips et al. 2014). Less common are records of pseudocopulation occurring in other monocotyledonous families. These include Amaryllidaceae (*Gilliesia graminea* Lindl.; Rudall et al. 2002) and Iridaceae (*Iris* Tourn. ex L.; Vereecken et al. 2012). It occurs, however, very rarely amongst eudicotyledonous families but is found in Combretaceae (*Guiera senegalensis* J.F. Gmel.; Kullenberg 1961) and Asteraceae (*Gorteria diffusa* Thunb.; Ellis and Johnson 2010). Insects are attracted to flowers of sexually deceptive species by both visual and olfactory signals (floral scents mimicking the sex pheromones of species-specific insect pollinators), as what occurs in orchids (e.g. Singer et al. 2004; Ascensao et al. 2005; Flach et al. 2006; Phillips et al. 2014) or solely by visual cues associated with sexual behaviour, as what occurs in eudicotyledonous *Gorteria* (De Jager and Ellis 2012). Indeed, *Gorteria diffusa* is possibly unique amongst sexually deceptive taxa in that it produces pollinator food rewards (Ellis and Johnson 2010).

If the pollination mechanism of *U. dunlopii* is indeed sexually deceptive, as has been proposed by Lowrie (1995), then its flowers should produce both visual and olfactory cues, e.g. possession of osmophores. Flowers of species assigned to the *U. capilliflora* complex are said to be slightly fragrant (Fleischmann 2010), but according to Jobson (2013), *U. lowriei* flowers do not emit an obvious fragrance perceptible to humans. The flower structure of *U. dunlopii* has already been well described by Taylor (1989) and Lowrie (2013), but both these studies lacked micromorphological details. Thus, the aim of the present paper is to compare the structure and distribution of glandular trichomes in *U. dunlopii* and to discuss their possible function in pseudocopulation. For comparative purposes, we selected species from the same section *Pleiochasia*, namely, *Utricularia paulinae* Lowrie, *Utricularia dichotoma* Labill. and *Utricularia uniflora* R.Br., that have ‘typical’ corolla structure (lower lip expanded to form wide platform, upper lip reduced; Taylor 1989; Lowrie 2013) and are not pseudocopulatory (for *U. dichotoma*; see Hingston and Mcquillan 2000).

## Material and methods

Species used in this study include *U. dunlopii* P.Taylor (living collections accession numbers ACCID 2011.01267 Botanická zahrada hl. m. Prahy, Czech Republic, and U65 Botanická zahrada Liberec, Czech Republic; herbarium material accession numbers MEL 576145 and MEL 653919 National Herbarium of Victoria (MEL) Melbourne, Australia), *U. paulinae* Lowrie (living collections accession number OBUJ54/2011 Jagiellonian University Botanical Garden), *U. dichotoma* Labill. (living collections accession number OBUJ06/2003 Jagiellonian University Botanical Garden) and *U. uniflora* R.Br. (living collections accession number OBUJ41/2010 Jagiellonian University Botanical Garden).

Voucher material of each of these species was deposited at the Department of Plant Cytology and Embryology, Jagiellonian University in Kraków.

The distribution of secretory glandular trichomes, unicellular hairs and papillae was determined by examining entire flowers under a stereoscopic microscope.

Floral parts with glandular trichomes were subsequently examined using light microscopy (LM), scanning electron microscopy (SEM) and transmission electron microscopy (TEM), as follows. Trichome-bearing floral parts were excised and fixed in 2.5 % (*v*/*v*) glutaraldehyde/4 % (*v*/*v*) formaldehyde in 0.1 M sodium cacodylate buffer (pH 7.0) for 2 h at 4 °C, washed three times in 0.1 M sodium cacodylate buffer pH and post-fixed in 1.5 % (*w*/*v*) osmium tetroxide solution for 1.5 h at 0 °C. Dehydration using a graded ethanol series, and infiltration and embedding using an epoxy embedding medium kit (Fluka) followed. Following polymerisation at 60 °C, sections were cut at 70 nm for TEM using a Leica ultracut UCT ultramicrotome, stained with uranyl acetate and lead citrate (Reynolds 1963) and examined using a Hitachi H500 transmission electron microscope at an accelerating voltage of 75 kV.

Semi-thin sections (0.9–1.0 μm thick) were prepared for LM and stained for general histology using aqueous methylene blue/azure II (MB/AII) for 1–2 min (Humphrey and Pittman 1974) and examined with an Olympus BX60 light microscope. The periodic acid–Schiff (PAS) reaction was also used to reveal the presence of insoluble polysaccharides, and Sudan Black B was used to detect the presence of lipids (Jensen 1962). Staining for total proteins was achieved using Coomassie brilliant blue R250 (Fisher 1968; Ruzin 1999) and mercuric bromophenol blue for total proteins (Mazia et al. 1953). For *U. paulinae*, hand-cut sections of floral tissues (petals and spur) were tested for lipids, starch and mucilage using a saturated ethanolic solution of Sudan III, aqueous IKI (iodine-potassium iodide) solution and ruthenium red solution, respectively (Ruzin 1999).

Nikon Eclipse E200 (NIS-Elements AR software) was used in conjunction with Nikon DS-Fi2 and a Canon D500 camera or an Olympus BX60 microscope for general photography and micrometry/photomicrography, respectively.

For SEM, the entire corolla or representative floral parts were dehydrated and subjected to critical-point drying using liquid CO_2_. They were then sputter-coated with gold and examined using a Hitachi S-4700 scanning electron microscope (Hitachi, Tokyo, Japan) based at the Scanning Microscopy Laboratory of the Department of Biological and Geological Sciences, Jagiellonian University in Kraków, at an accelerating voltage of 20 kV.

## Results

### *U. dunlopii*

**Fig. 1:**
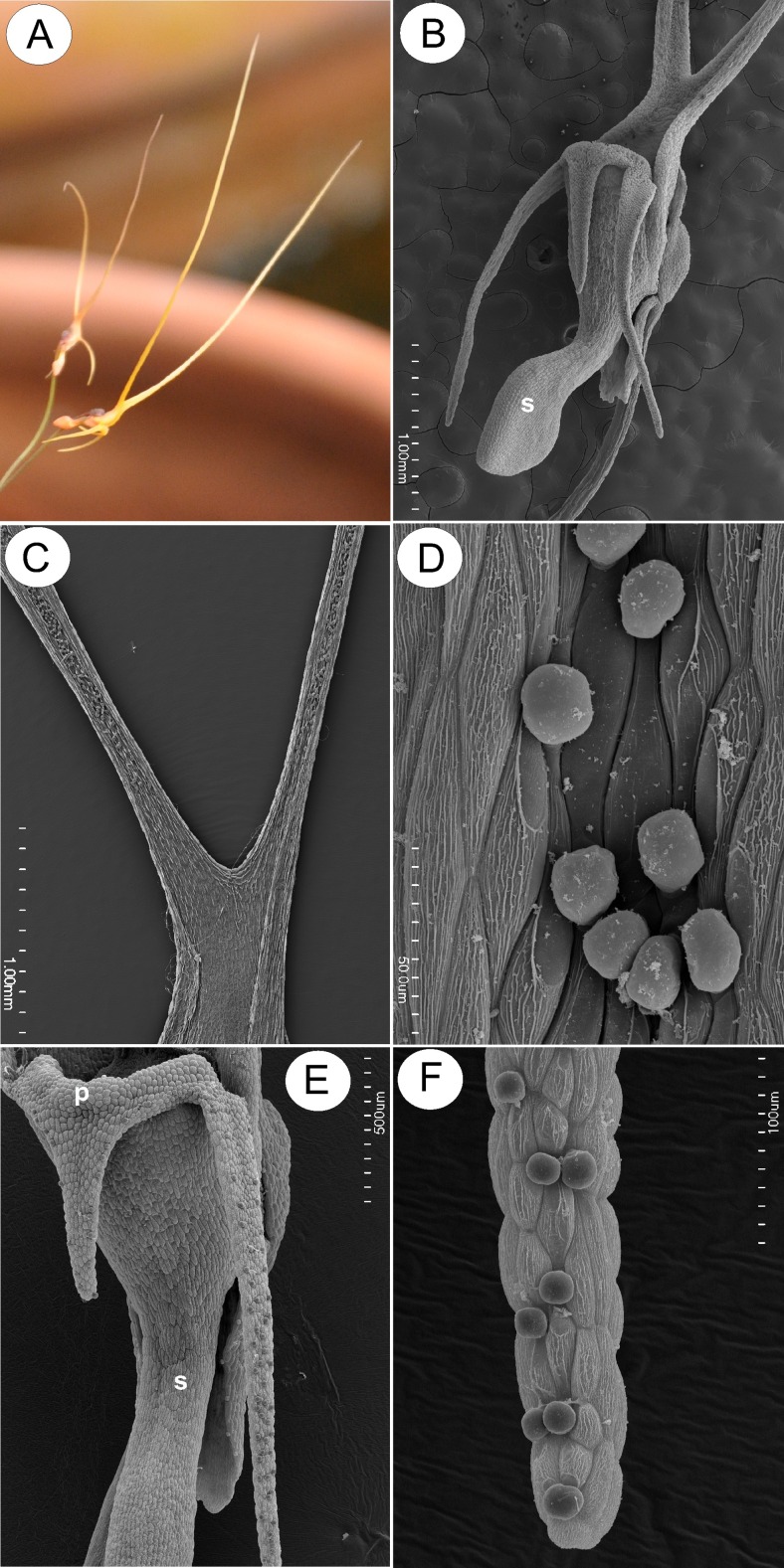
General morphology of *Utricularia dunlopii* flowers; **a** two-flowered inflorescence. Plant in culture in Liberec Botanical Garden. **b** General morphology of flower under SEM; note the bursiform spur (*s*); *scale bar* = 1 mm. **c** Upper corolla lip consisting of two fused petals, each bearing a single corolla appendage with shallow adaxial groove containing glandular hairs; material from herbarium (MEL); *scale bar* = 1 mm. **d** Part of upper corolla lip appendage with numerous glandular hairs; *scale bar* = 500 μm. **e** The palate (*p*), appendages of lower corolla lip with glandular trichomes and spur (*s*); *scale bar* = 500 μm. **f** Part of lower corolla lip appendage with numerous glandular hairs; *scale bar* = 100 μm

The corolla of *U. dunlopii* is pale orange-brown in colour. The upper lip of the corolla comprises two fused petals, each having a long, erect, filiform appendage or extension, whereas the lower lip consists of three fused petals with short lobes (Fig. 1a–c). The lower corolla lip forms a small bursiform spur (Fig. 1b), unlike that of any related species.

In *U. dunlopii*, the adaxial surface of the upper corolla lip was covered with glandular trichomes. These also occurred on all parts of the adaxial surface of the upper corolla lip appendages, except for their bases (Fig. 1 c, d). The appendages were shallowly grooved, the grooves containing glandular trichomes and epidermal cells with a smooth cuticle. By contrast, the cuticle overlying the external wall of marginal epidermal cells had fine, perpendicular striations (Fig. 1d). No droplets of secretion were visible on appendages in living material.

**Fig. 2:**
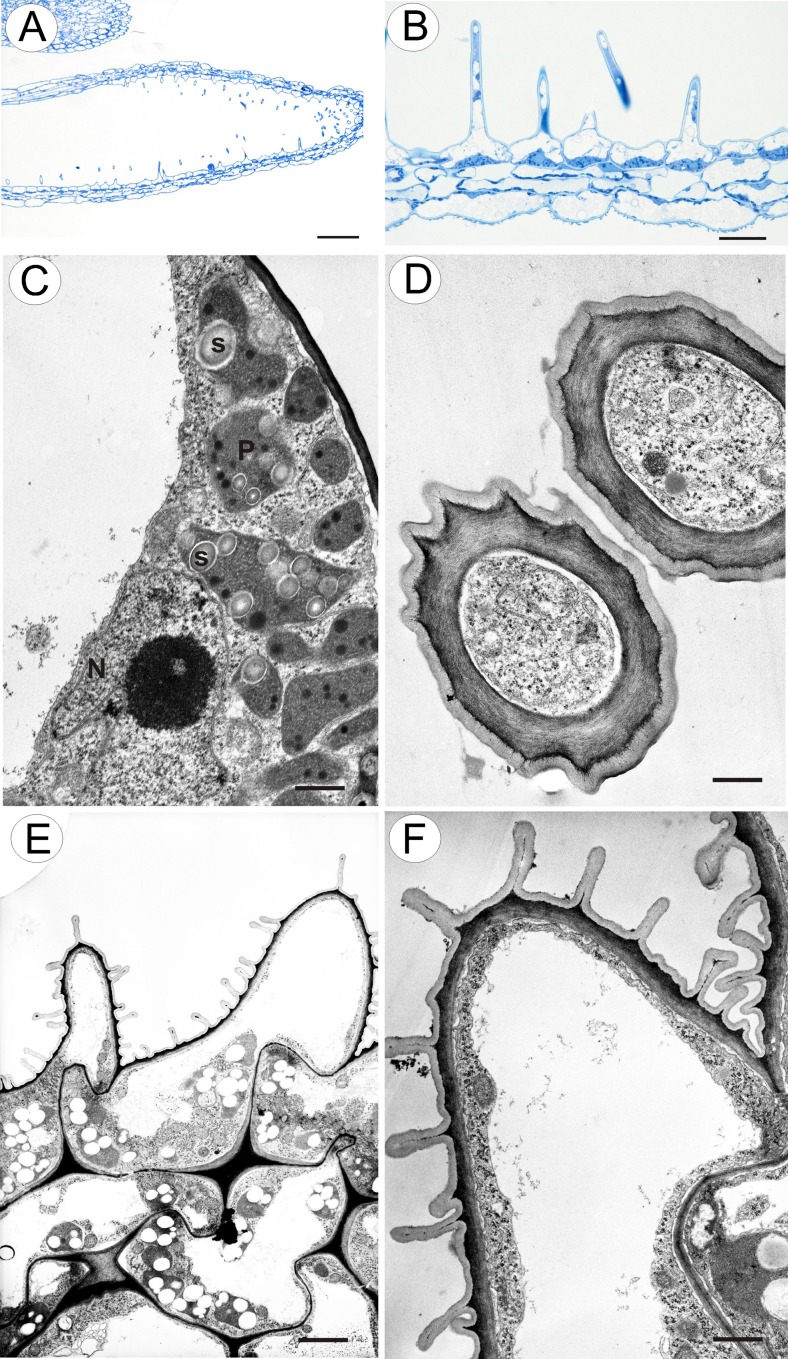
Structure of the spur and upper corolla lip appendages of *Utricularia dunlopii* flowers. **a** Longitudinal section through the spur; *scale bar* = 50 μm. **b** Spur papillae projecting into lumen; *scale bar* = 20 μm. **c** Ultrastructure of base of papilla showing plastid (*P*), starch (*S*) and nucleus (*N*); *scale bar* = 0.85 μm. **d** Transverse section through papillae showing thick cuticle with numerous microchannels; *scale bar* = 0.80 μm. **e**, **f** Epidermis of upper corolla lip appendage. Note the striate cuticle; *scale bar* = 2.5 and 0.83 μm, respectively

Glandular trichomes were absent from the epidermis lining the entrance to the lumen of the spur. The corolla palate formed a glabrous ring of tissue (Fig. 1e), and the cuticle overlying its epidermal cells was obviously striate. Trichomes were also present on the margins of the central lobe (petal) of the lower lip of the corolla, and the entire adaxial surface of the lateral lobes (Fig. 1e, f). Glandular trichomes also occurred on the margins of the calyx and were scattered amongst unicellular papillae within the spur (Fig. 2a–d). The papillae had evident cuticular striations (Fig. 2d) and were highly vacuolate. Cytoplasm with nucleus and plastids were mainly located in the basal part of the papillae (Fig. 2c). TEM observations revealed that the cuticle contained numerous microchannels (Fig. 2d).

**Fig. 3:**
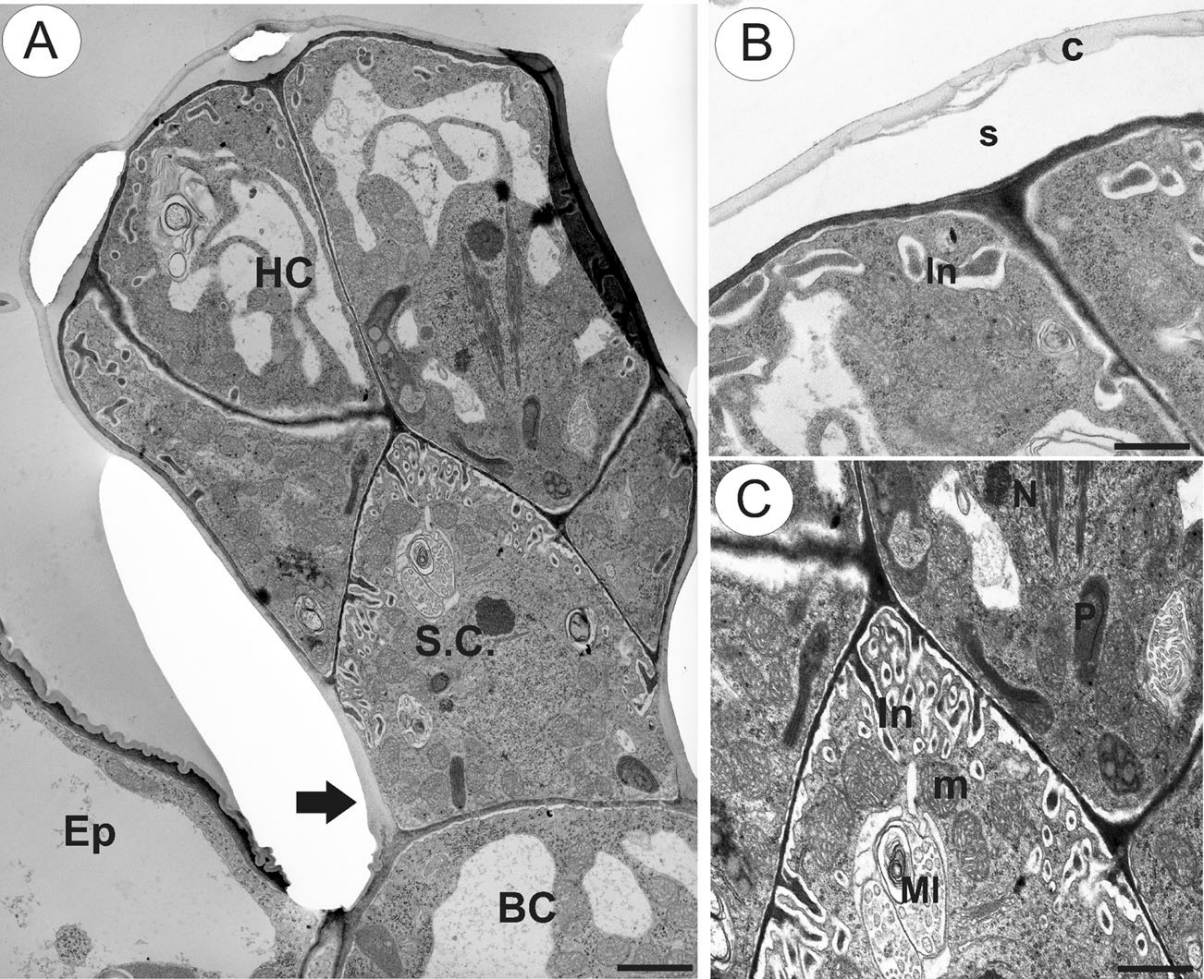
Ultrastructure of glandular trichome (osmophore) from corolla lip appendages of *Utricularia dunlopii* flowers; **a** longitudinal section showing head cells (*HC*), stalk cell (*SC*), basal cell (*BC*), thickened anticlinal wall of stalk cell (*arrow*) and epidermal cell (*Ep*); *scale bar* = 1.6 μm. **b** Section through head cells showing cuticle (*c*), subcuticular space (*s*) and cell wall ingrowths (*In*); *scale bar* = 0.7 μm. **c** Section through head cells and stalk cell showing mitochondria (*m*), plastid (*P*) and nucleus (*N*). Note the well-developed wall ingrowths in stalk cell (*In*) and myelin-like body (*MI*); *scale bar* = 0.9 μm

SEM observations did not reveal the presence of secretion on the surface of head cells of glandular trichomes located on the corolla lobe (Fig. 1d). Regardless of the location of glandular trichomes, the cuticle overlying the head cells was smooth and intact, without visible pores or cracks. Similarly, the cuticle covering the epidermal cells of filiform corolla appendages was also smooth and intact, with some electron-dense surface material visible under TEM (Fig. 2e, f). Generally, glandular trichomes present on the surface of the flowers of *U. dunlopii* were composed of a single basal cell, a unicellular stalk and a four-celled head (Fig. 3a).

**Fig. 4:**
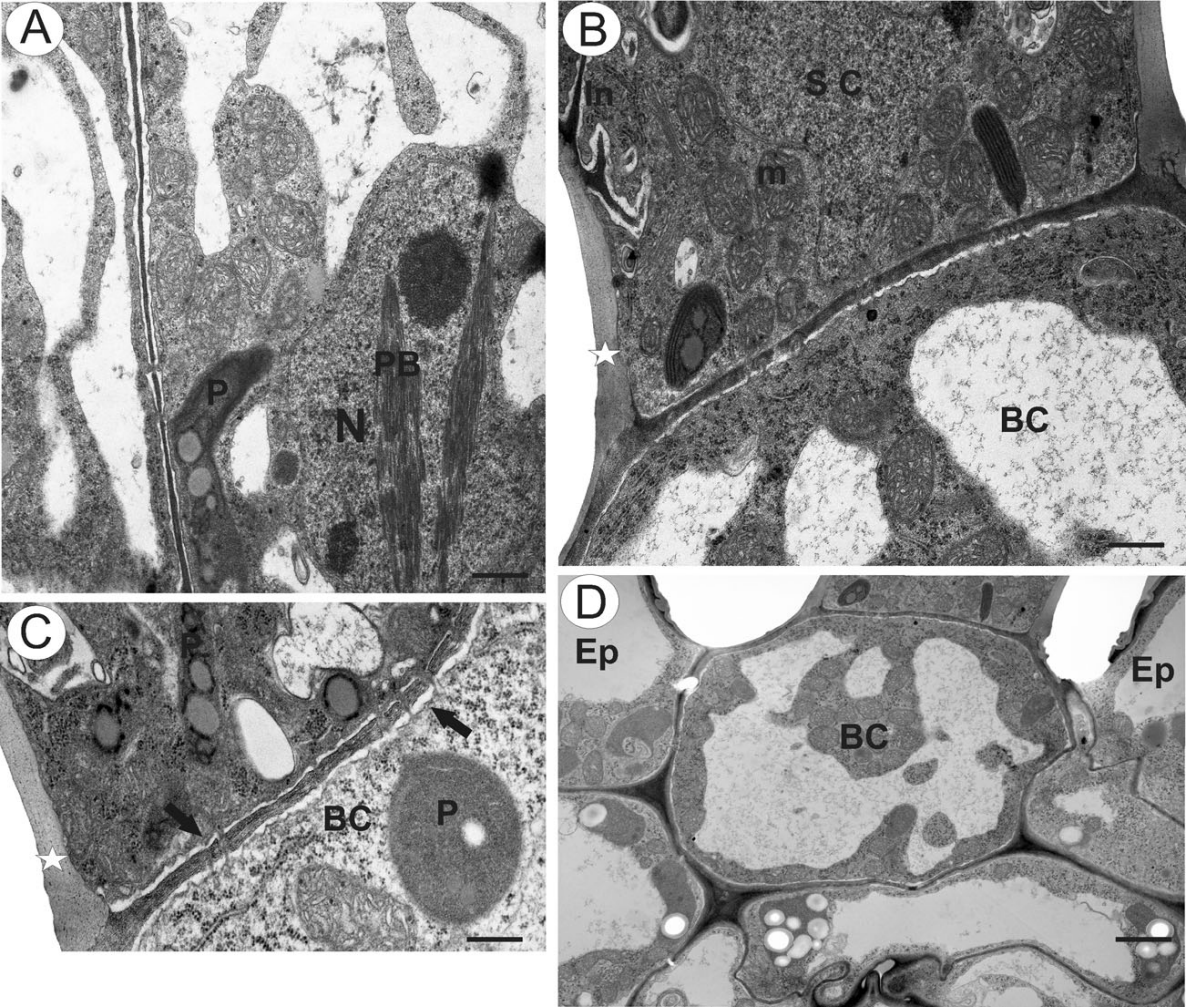
Ultrastructure of glandular trichome from upper corolla lip appendages of *Utricularia dunlopii* flowers; **a** section through head cells. Note the intranuclear paracrystalline body (*PB*) and the presence of numerous mitochondria in the cytoplasm, plastid (*P*) and nucleus (*N*); *scale bar* = 0.55 μm. **b**, **c** Numerous plasmodesmata (*arrow*) in transverse cell wall between stalk cell (*SC*) and basal cell (*BC*). Note the basally thickened anticlinal wall of the stalk cell, wall ingrowths (*In*) and mitochondria (*m*); *scale bar* = 0.6 and 0.5 μm. **d** Ultrastructure of basal cell (*BC*) and epidermal cell (*Ep*); *scale bar* = 1.9 μm

TEM observations revealed that the cuticle frequently became distended and separated from the cell walls of the head cells (Fig. 3a, b). Ingrowths (Fig. 3b) arose from the inner surface of the outer walls of head cells (Fig. 3b), and similar structures projected into the cytoplasm of the stalk cell (Fig. 3c). Furthermore, primary pit fields with plasmodesmata were present in anticlinal walls of head cells (Fig. 4a) and in the tangential walls between stalk and head cells, and between the stalk and basal cell of the trichome (Fig. 4b, c). The protoplasts of head cells were electron-dense, with a prominent, centrally located nucleus containing a paracrystalline protein inclusion (Figs. 3a and 4a). Scattered elongate plastids, each containing an electron-dense stroma and peripheral thylakoids, together with large lipid globules, were present (Fig. 4a). Similar plastids were also present in stalk cells (Fig. 3c). Individual, small lipid droplets were also visible in the cytoplasm. Mitochondria, long profiles of rough endoplasmic reticulum (RER), dictyosomes and secretory vesicles were common in the cytoplasm (Figs. 3a, c and 4a). Although RER and secretory vesicles are collected in the parietal cytoplasm alongside the plasmalemma, stages in the fusion of vesicles with the plasmalemma and the presence of vesicles in the periplasmic space were not observed. Intravacuolar myelin-like figures and variously sized globular or flocculent electron-dense material (Fig. 3a, c) were present in both head and stalk cell. The anticlinal wall of the stalk cell was thickened at its base, where it came into contact with the periclinal wall of the basal cell (Fig. 4b, c). In contrast to the head and stalk cells, the basal cell was highly vacuolate (Fig. 4d).

### Histochemical analysis of upper corolla appendages of *U. dunlopii*

**Fig. 5:**
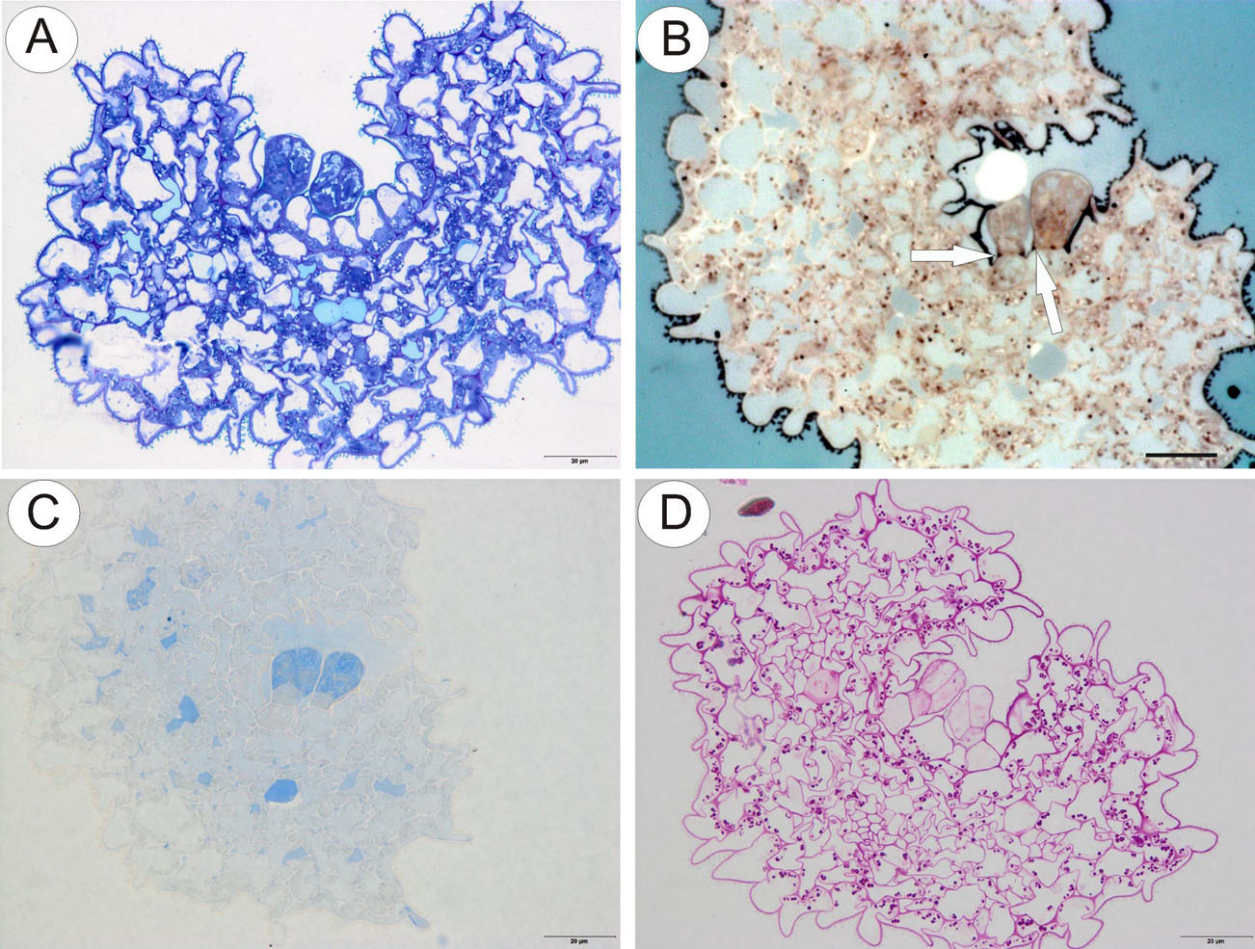
Histochemical analysis of glandular trichomes from upper corolla lip appendages of *Utricularia dunlopii* flowers; **a** section of upper corolla lip appendage stained with methylene blue/azure II for general histology; *scale bar* = 20 μm. **b** The lipid stain SBB was taken up selectively by basal parts of the anticlinal cell walls of the stalk cell (*arrow*); *scale bar* = 20 μm. **c** Protein stained with mercuric bromophenol blue; *scale bar* = 20 μm. **d** Starch was present in both epidermal and parenchyma cells of corolla appendages but absent from the cells of glandular trichomes (PAS reaction); *scale bar* = 20 μm

The cytoplasm of both head and stalk cells of the glandular trichome stained deeply with methylene blue/azure II (Fig. 5a). Lipid droplets were not visible in the cytoplasm of head cells using LM, but Sudan III and SBB stained the cuticle at the base of the anticlinal walls of stalk cells, and lipid droplets in the plastids of parenchyma cells, intensely (Fig. 5b). The striate cuticle overlying the epidermal cells also stained with SBB, but the cuticle of the head cells of glandular trichomes did not stain with this reagent, indicating possible differences in cuticular permeability, thus facilitating secretion. Furthermore, although Coomassie brilliant blue did not generally stain the tissues of the corolla appendages, the rather stronger staining of the head cells of glandular trichomes with this reagent indicated that they contained elevated concentrations of proteinaceous material (Fig. 5c). Histochemical tests using IKI and the PAS reaction revealed only minute quantities of starch in the head cells of glandular trichomes of *U. dunlopii*, in contrast to corolla appendage epidermal and parenchyma cells, which accumulated starch grains (Fig. 5d).

### Other species

**Fig. 6:**
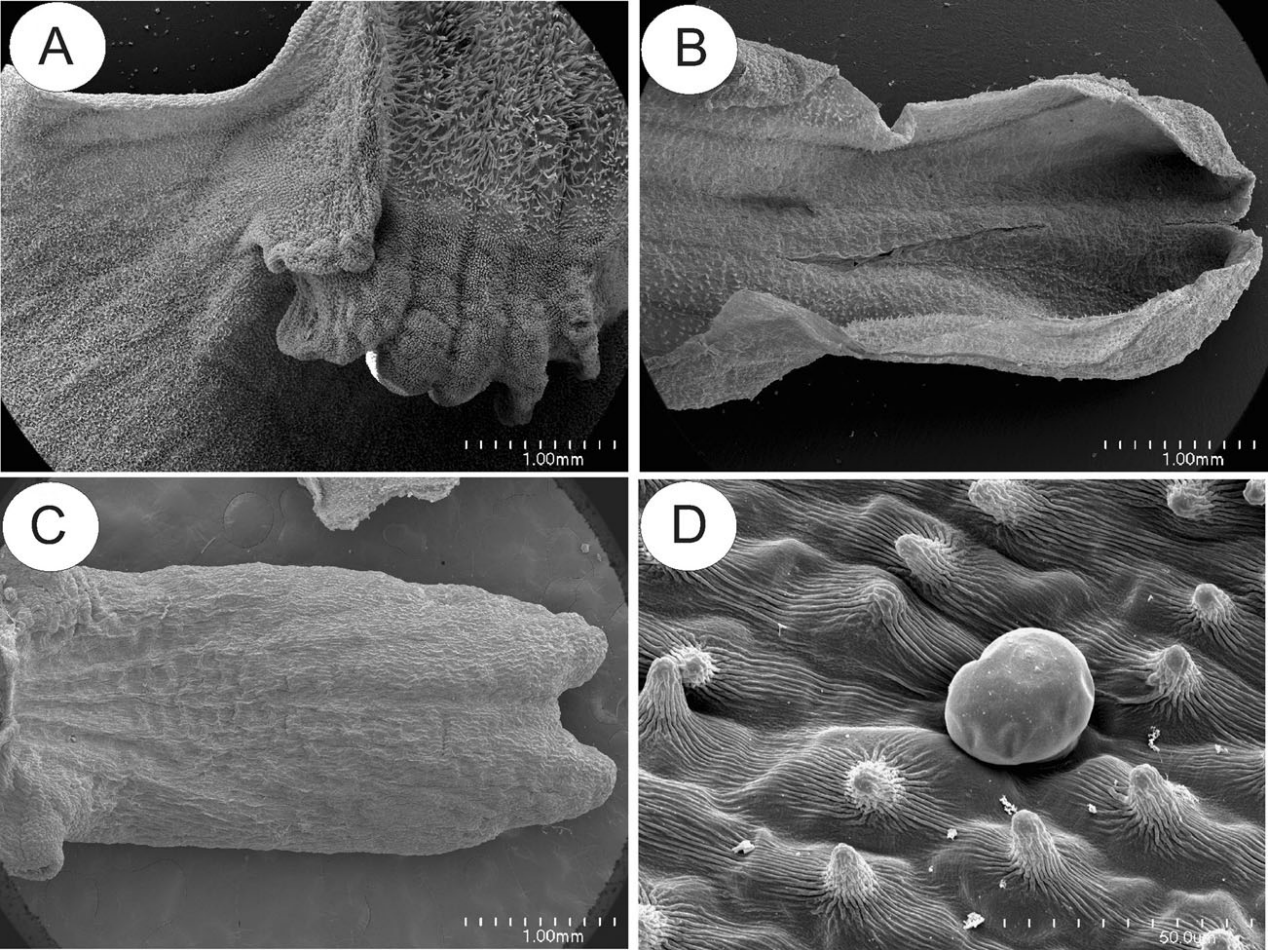
Micromorphology of *U. uniflora* and *U. dichotoma* flowers; **a** lower lip of *U. uniflora.* Note the well-developed corolla palate; *scale bar* = 1 mm. **b** Spur of *U. uniflora*; *scale bar* = 1 mm. **c**, **d** Spur of *U. dichotoma* lined with glandular trichomes; *scale bar* = 1 mm and 50 μm, respectively

**Fig. 7:**
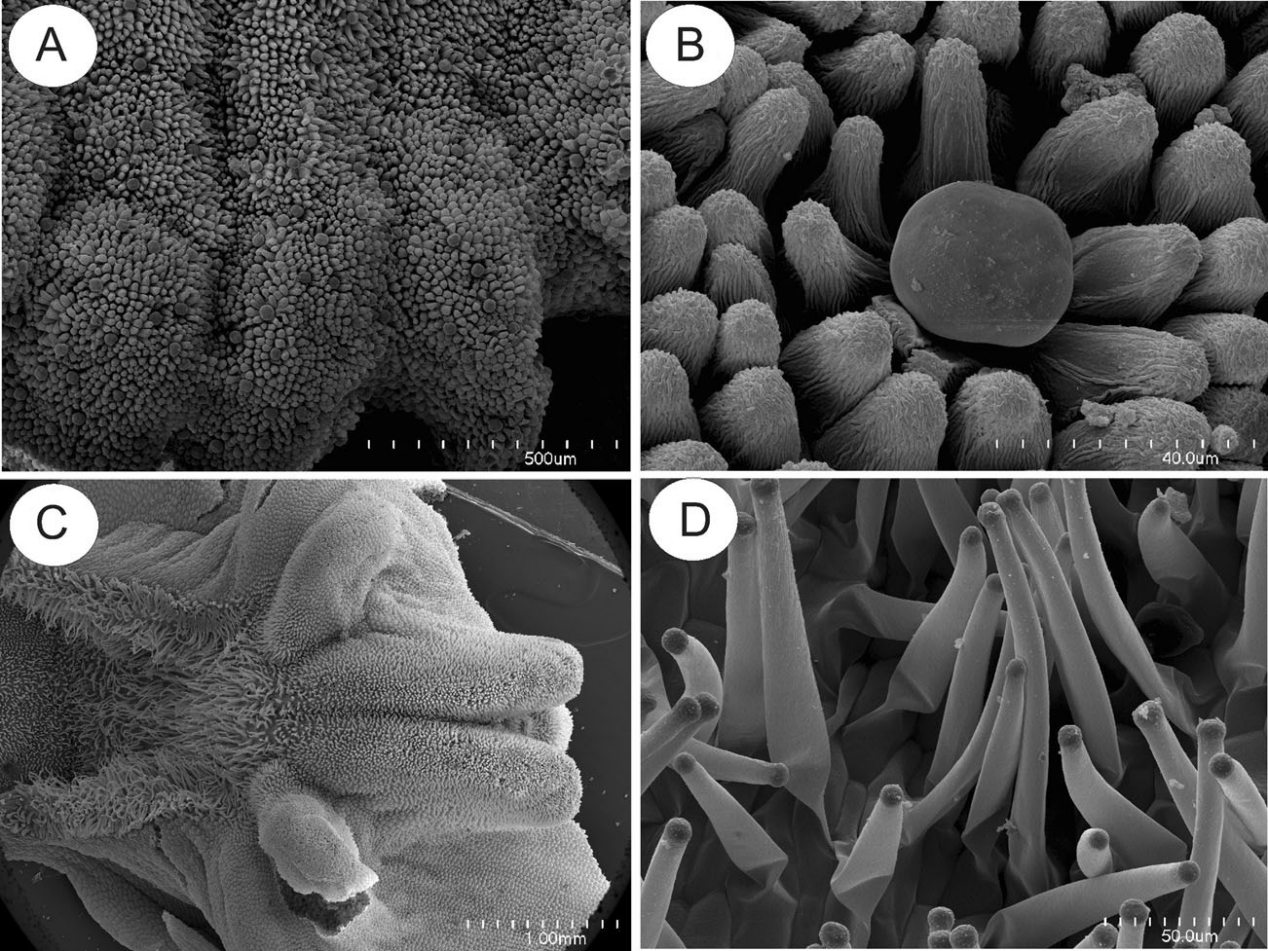
Micromorphology of corolla palate of *U. uniflora* and *U. dichotoma*; **a** corolla palate of *U. uniflora* with numerous glandular trichomes; *scale bar* = 500 μm. **b** Glandular trichome on corolla palate of *U. uniflora.* Note arrangement of papillae surrounding glandular trichome; *scale bar* = 40 μm. **c** Corolla palate of *U. dichotoma*; *scale bar* = 1 mm. **d** Unicellular, somewhat capitate papillae of *U. uniflora* with spherical tips located on area between corolla palate and spur; *scale bar* = 50 μm

In *U. paulinae*, *U. dichotoma* and *U. uniflora*, the upper lip of the corolla lacks filiform appendages and is reduced, in contrast to the lower lip which forms a wide platform (Fig. 6a). In these three species, the floral spur is cylindrical (Fig. 6b, c), and the palate, papillose (Fig. 7a–c). Glandular trichomes of similar structure to those of *U. dunlopii*, and composed of a single basal cell, a unicellular stalk and a four-celled head (Fig. 6a), were present on the surface of the corolla of the remaining species investigated (Figs. 6d and 7a, b). However, these trichomes were distributed differently depending on the species. In *U. uniflora* and *U. paulinae*, glandular trichomes occurred on the surface of the corolla palate (Fig. 7a, b), in contrast to *U. dichotoma* (Fig. 7c), where they were absent. In the floral spurs of *U. dichotoma* and *U. paulinae*, glandular trichomes were interspersed amongst small papillae (Fig. 6d), whereas in *U. dichotoma*, glandular trichomes also occurred on the external spur surface, together with small droplets of secretion. The epidermis lining the inner surface of the spur of *U. uniflora* consisted entirely of unicellular papillae. SEM observations of the corolla glandular trichomes of *U. uniflora* revealed the presence of secretion on the surface of head cells (Fig. 7b). Secretory material was also present on the surface of head cells of trichomes lining the spur of *U. dichotoma*. Regardless of species and location of the trichome, the cuticle overlying trichomal head cells was smooth and intact, without visible pores or cracks. Conversely, the cuticle overlying epidermal cells and unicellular papillae located within the spurs was striate, and no secretory material was observed (Fig. 6d). Long, unicellular, capitate papillae with spherical tips (Fig. 7d) were present in *U. paulinae*, *U. dichotoma* and *U. uniflora.*

### Histochemical analysis of *U. paulinae*

Treatment of *U. paulinae* with IKI and PAS reagents revealed only minute quantities of starch in the head cells of the glandular trichomes of *U. paulinae*, but starch grains were more frequent in the epidermal and parenchyma cells of the corolla lobe. Lipids were also absent from the head cells of trichomes, and again, Sudan III and SBB stained the basal parts of anticlinal walls of stalk cells. Furthermore, in *U. paulinae*, staining of hand-cut sections with ruthenium red solution did not reveal the presence of mucilage, nor did staining with Coomassie brilliant blue indicate the accumulation of proteinaceous material.

## Discussion

Sexually deceptive plant taxa generally possess floral characters adapted to pollination by highly specific pollinators. The flower is often insect-like, lacks food rewards and tends to produce fragrances that chemically mimic pheromones produced by females of the species of insect involved in its pollination (Phillips et al. 2014). The most convincing evidence for pseudocopulation, however, is the pre-mating behaviour of male insects at the flower and their attempted copulation with it. For *U. dunlopii*, as well as other species assigned to the *U. capilliflora* complex, there is a lack of published data relating to this phenomenon, merely conjectures. Taylor (1989) suggested, based solely on flower colour, that *U. dunlopii* and its allies may be pollinated by dipterans (flies). Likewise, the proposal made by Lowrie (1995) for sexual deception as the means of pollination in this species was based on both the specific colour and the three-dimensional form of the flower.

Other features that occur in pseudocopulatory and presumed pseudocopulatory orchid taxa are metallic blue, reflective epidermal surfaces and long, narrow, unicellular trichomes with pointed tips and narrow points of insertion. Such reflective surfaces are represented by the labellar speculum of species of *Ophrys* L. (Servettaz et al. 1994; Ascensao et al. 2005), but have also been observed for *Mormolyca schweinfurthiana* Garay & Wirth (Davies and Stpiczyńska 2006), and apically on the petals of *Trigonidium aurorae* D. E. Benn. & Christenson and *Trigonidium egertonianum* Bateman ex Lindl. (Whitten and Blanco 2011), as well as on the sepals of *Trigonidium obtusum* Lindl. (Singer 2002), whereas this particular type of trichome occurs on the labella of *Mormolyca ringens* (Lindl.) Gentil, *M. schweinfurthiana* (Davies and Stpiczyńska 2006) and *Ophrys* spp. (Servettaz et al. 1994; Ascensao et al. 2005). Long unicellular hairs, although not identical to those described above, were also present on the surface of the palate of *U. paulinae*, *U. dichotoma* and *U. uniflora.*

Flowers of most sexually deceptive taxa, like many others, also have osmophores (Phillips et al. 2014). However, in the past, this term has been applied both to the scent glands (e.g. Vogel 1990), as used in the present paper, and to the structure that bears them (e.g. Pridgeon and Stern 1983, 1985), which in the present case is the petal appendages. According to Vogel (1990), the osmophore (scent gland) is a specialised flower organ or glandular trichome that produces fragrance. In order to distinguish between these completely different, yet related structures, we propose that the term osmophore be reserved for fragrance-producing epidermal structures, such as glands, and that an alternative term (*unguentarius* Latin = perfumer) be adopted for the organ that bears them. This organ is frequently a morphologically modified petal or sepal and bears secretory hairs, glands or glabrous epithelium. We believe that the corolla appendages of *U. dunlopii* fulfil this role, especially as they bear glandular trichomes and are similar in appearance and organisation to the antenniform, osmophore-bearing petals and sepals of certain orchids, such as species of *Bulbophyllum* Thouars, *Diplodium* Sw, *Restrepia* Kunth and *Scaphosepalum* Pfitzer (Pridgeon and Stern 1983, 1985; Vogel 1990; Kowalkowska et al. 2015), as well as *Gilliesia graminea* (Amaryllidaceae J.St.-Hil.; Rudall et al. 2002). In sexually deceptive taxa, the osmophores (scent glands) secrete substances which mimic the sex pheromone of the pollinator, and this is well documented for orchids, e.g. *Ophrys* (Schiestl et al. 1999; 2000), *Chiloglottis* R.Br. (Schiestl et al. 2003), *Cryptostylis* R.Br. (Schiestl et al. 2004), *Mormolyca* Fenzl. (Singer et al. 2004; Flach et al. 2006) and *Trigonidium* Lindl. (Singer 2002). Unfortunately, many non-pseudocopulatory taxa also possess osmophores, some of which secrete fragrances not perceptible to humans.

Histochemical and structural data concerning osmophores in sexually deceptive orchids are relatively scarce, and most relate to several species of European *Ophrys* (Servettaz et al. 1994; Ascensao et al. 2005; Francisco and Ascensão 2013). Here, the papillose osmophore contained numerous cytoplasmic lipid droplets, with starch grains occurring in both epidermal and parenchyma cells. Similar results were also obtained for the osmophores of other orchid genera and species, such as *Bulbophyllum* (Teixeira et al. 2004; Kowalkowska et al. 2015), *Chloraea membranacea* Lindl. (Sanguinetti et al. 2012), *Cyclopogon elatus* (Sw.) Schltr. (Wiemer et al. 2009), *Cycnoches* Lindl. (Antoń et al. 2012), *Restrepia* Kunth (Pridgeon and Stern 1983), *Scaphosepalum* Pfitzer (Pridgeon and Stern 1985) and *Stanhopea* J. Frost ex Hook. (Stern et al. 1987; Antoń et al. 2012). However, lipid was scarce in the cytosol of the head cells of the glandular trichomes of *U. dunlopii.* Instead, these cells contained large lipid globules within elongate plastids that were surrounded by numerous ER profiles, possibly indicating fragrance (terpenoid) synthesis (Lange and Turner 2013). Secretion, here, may be eccrine, and the presence of wall ingrowths in head cells of glandular trichomes suggests that they may function as transfer cells. Indeed, wall ingrowths are a common feature of the glandular trichomes of Lentibulariaceae, having already been demonstrated to occur in the traps of these carnivorous plants (e.g. Fineran 1985; Płachno et al. 2007).

It should, however, also be stressed that osmophores occur in many taxa which are not pollinated by sexual deceit, for example many orchids (e.g. Stpiczyńska, 1993, 2001) and in stapeliads that display carrion mimicry (e.g. Jürgens et al. 2006; Meve and Liede 1994; Płachno et al. 2010).

In *U. uniflora* and *U. paulinae*, the corolla palate may also fulfil the role of an unguentarius, since it bears glandular trichomes that do not produce nectar, the floral spurs of these species being larger and nectariferous. Therefore, we cannot exclude the possibility that the corolla palate represents a transitional evolutionary step between the formation of a landing platform and unguentarius, the pollinators being attracted mainly to floral nectar. In *U. dunlopii*, the nectary spur is reduced, and pollinators are attracted largely by the insectiform configuration of the flower and volatilisation of fragrance produced by glandular trichomes (osmophores) densely distributed upon the modified floral appendages or unguentari.

A very important floral feature of sexually deceptive species is the insectiform habit. Although its distribution is restricted mainly to sexually deceptive orchids, it is also known to occur elsewhere, e.g. *Gilliesia graminea* Lindl. (Amaryllidaceae; Rudall et al. 2002). We agree with Lowrie’s (1995) observation that the flower of *U. dunlopii* resembles an insect, but are mindful of the *caveat* that insectiform or arachniform flowers can also be the subjects of other possible pollination syndromes, including pseudocarnivory or pseudoparasitism, as occur in some orchid species (Christensen 1994).

The floral anatomical and morphological organisation of *U. dunlopii* differs from that of other species investigated to date and assigned to the same section, thus strongly indicating that this species has a different insect pollinator to the rest.

Many of the floral characters found in *U. dunlopii* also occur in other species of the *U. capilliflora* complex. All these species (*U. capilliflora*, *U. dunstaniae*, *U. antennifera* and *U. lowriei*) have an insectiform flower, a reduced spur and an apricot- or flesh-coloured corolla (Taylor 1989; Lowrie 1995, 2013; Reut and Jobson 2010; Jobson 2013), thus strongly suggesting that they have the same or a similar pollinator to *U. dunlopii*. Further study will show whether they also possess glandular trichomes on the filiform appendages of the corolla. Another species of *Utricularia* having a reduced spur and minutely glandular, filiform appendages to the corolla was recently described (Jobson and Baleeiro 2015). Remarkably, however, in contrast to the aforementioned taxa, *Utricularia wannanii* R.W. Jobson & Baleeiro has a white corolla and also differs in its ecology. For example, many members of the *U. capilliflora* complex prefer habitats which are frequently shallowly flooded during the wet season (Lowrie 1995, 2013; Jobson 2013), whereas *U. wannanii* has a lithophytic habit (Jobson and Baleeiro 2015).

As previously stated, most species of the *U. capilliflora* complex have similar ecology and a relationship exists between the ecological niche inhabited by these taxa, and their specific mode of pollination cannot be dismissed. Jobson (2013) found *U. lowriei* growing together with several other *Utricularia* species, namely, *Utricularia albiflora* R.Br., *Utricularia caerulea* L., *Utricularia chrysantha* R.Br., *Utricularia subulata* L., *Utricularia quinquedentata* F.Muell. ex. P. Taylor and *Utricularia gibba* L. It is thus possible that competition for the same or similar pollinators in a niche inhabited by many other species of *Utricularia* drives evolutionary changes such as modification of floral form and specialisation of pollination strategies.

Our morphological and micromorphological investigations, while providing further information about the general structure of the flower of *U. dunlopii*, did not refute the possibility that pollination in this species may indeed occur by pseudocopulation. Only field-based observations of the pollination process will, however, confirm that this is unequivocally so. In order to understand more fully the evolution and pollination of the flower of *U. dunlopii* and related taxa, future studies should address the composition of floral fragrances, including analysis by means of solid phase microextraction and gas chromatography coupled with mass spectrometry.
